# The efficacy of high-frequency repetitive transcranial magnetic stimulation on upper extremity function in intracerebral hemorrhage: a real-world retrospective cohort study

**DOI:** 10.3389/fneur.2025.1683536

**Published:** 2025-12-10

**Authors:** Shasha Fan, Junyan Zhang, Yajuan Li, Xiangping Li, Qing Niu, Ke Zhang, Pingzhi Wang, Xiaolei Liu

**Affiliations:** 1 Department of Rehabilitation Medicine, Shanxi Bethune Hospital, Shanxi Academy of Medical Sciences, Tongji Shanxi Hospital, Third Hospital of Shanxi Medical University, Taiyuan, China; 2 Department of Clinical Epidemiology and Evidence-based Medicine, Shanxi Bethune Hospital, Shanxi Academy of Medical Sciences, Tongji Shanxi Hospital, Third Hospital of Shanxi Medical University, Taiyuan, China; 3 Department of Rehabilitation Medicine, First Hospital of Shanxi Medical University, Taiyuan, China; 4 Department of Neurology, The First Affiliated Hospital of Kunming Medical University, Kunming, China

**Keywords:** retrospective cohort, repetitive transcranial magnetic stimulation, stroke, intracerebral hemorrhage, Fugl-Meyer upper extremity scale

## Abstract

**Background:**

Repetitive transcranial magnetic stimulation (rTMS) has been applied to improve upper limb motor recovery following stroke. However, most existing evidence is derived from ischemic stroke populations, with limited studies specifically evaluating its efficacy in intracerebral hemorrhage (ICH).

**Objective:**

To explore the efficacy of rTMS on upper extremity function in a cohort exclusively composed of ICH patients, providing real-world evidence on its application in this underrepresented population.

**Methods:**

A retrospective cohort study was conducted including 394 patients diagnosed with ICH who were admitted to the Department of Rehabilitation Medicine at Bethune Hospital of Shanxi between January 2020 and June 2023. Patients were divided into two groups: conventional rehabilitation therapy alone or in combination with high-frequency rTMS (HF-rTMS). The primary endpoint was the proportion of patients achieving a clinically meaningful improvement (≥9 points) in the Fugl-Meyer Upper Extremity (FM-UE) Scale.

**Results:**

HF-rTMS treatment was significantly associated with an increased likelihood of achieving the primary endpoint (OR = 3.19, 95% CI 1.81–5.65, *p* < 0.001). Consistent findings were observed in the Overlap-Weighted PSM dataset, where a significantly higher percentage of participants in the HF-rTMS group met the primary endpoint compared to the non-rTMS group (35.29% vs. 20.60%, *p* = 0.003) confirming the robustness of our results.

**Conclusion:**

This large-scale real-world study exclusively focused on ICH provides critical evidence supporting the efficacy of HF-rTMS in subacute ICH rehabilitation. Given the paucity of dedicated research on ICH, these findings offer valuable insights into personalized neuromodulatory treatment strategies for this understudied population.

## Introduction

Despite understanding and medical conditions have improved, stroke remains the leading cause of disability-adjusted life years (DALYs) globally (1). Upper limb motor dysfunction, a common sequela, is still present in 50 to 70% of survivors 6 months after onset, leading to permanent motor impairment (2). Such functional impairments not only severely affect the quality of life of patients but also significantly increase the economic burden on families and society (3), highlighting the urgent need to develop effective rehabilitation interventions.

According to the interhemispheric inhibition imbalance theory, after a stroke, the hyperexcitability of the unaffected hemisphere suppresses the functional recovery of the affected hemisphere via transcallosal inhibition (4). Repetitive transcranial magnetic stimulation (rTMS) can improve this imbalance through frequency-dependent modulation: high-frequency repetitive transcranial magnetic stimulation (HF-rTMS) applied to the affected hemisphere can enhance cortical excitability, while low-frequency repetitive transcranial magnetic stimulation (LF-rTMS) applied to the healthy hemisphere can reduce its excitability, thereby promoting the recovery of upper limb motor function (5). Mechanistically, rTMS modulates the neuronal excitation–inhibition equilibrium, promotes synaptic strengthening through long-term potentiation (LTP), and enhances neuroplasticity by inducing the release of neurotrophic factors (6).

The therapeutic efficacy of rTMS is highly time-dependent. The subacute phase, spanning approximately 1 week to 6 months after stroke onset, is recognized as the “golden window” for neurorehabilitation, characterized by enhanced neuronal plasticity and spontaneous repair processes. In this phase, heightened endogenous repair mechanisms, such as neurogenesis and synaptic reorganization, can interact synergistically with rTMS-induced cortical modulation, thereby optimizing functional recovery (7). However, the 2020 international guidelines reported Level A evidence for low-frequency rTMS applied to the contralesional M1 in treating hand dysfunction after intracerebral hemorrhage (ICH), whereas high-frequency rTMS applied to the ipsilesional M1 was supported only by Level B evidence (5). Notably, the majority of available evidence originates from ischemic stroke populations, whereas data on ICH remain extremely scarce. In fact, patients with ICH constituted less than 5% of the 42 studies cited in the guidelines (5). This imbalance in evidence has fueled ongoing debate over the clinical use of rTMS in ICH and contributed to its omission from stroke management guidelines in many countries. One major reason is the inconsistency of existing findings (8–10), largely due to the wide variability in effect sizes reported by small proof-of-concept trials (11). Therefore, large-scale real-world studies are essential to delineate the therapeutic scope and determinants of HF-rTMS in patients with subacute ICH, thereby advancing personalized neuromodulatory interventions.

Using a real-world medical database, this presented study established a retrospective cohort of patients with subacute ICH to address two key questions: (1) whether 10 Hz HF-rTMS applied to the lesioned M1 can significantly improve upper limb motor function, with changes in the Fugl-Meyer Assessment serving as the primary endpoint; and (2) which patient subgroups (e.g., hemorrhage location, onset-to-intervention interval, baseline Fugl-Meyer Upper Extremity score) may derive the greatest therapeutic benefit. To our knowledge, this is the first study to systematically evaluate the efficacy of high-frequency stimulation in an ICH population, providing important evidence to inform future guideline development.

## Materials and methods

### Study design and participants

This retrospective cohort study aimed to evaluate the effectiveness of HF-rTMS in patients with ICH. From January 2020 to June 2023, 731 patients with ICH were hospitalized, of whom 394 participants were enrolled, with 221 receiving HF-rTMS (HF-rTMS group), and the remaining 173 who did not undergo HF-rTMS comprised the non-rTMS group.

Participants were enrolled if they met all of the following conditions:

- Hospitalized for initial treatment of ICH in the Department of Rehabilitation Medicine at Shanxi Bethune Hospital.
- Met the diagnostic criteria for ICH as outlined in the guideline (11).
- Had a symptom duration of 1 week to 6 months, corresponding to the subacute stage according to the 2020 evidence-based guidelines (5).
- Able to undergo rehabilitation therapy.
- Experienced their first stroke with a unilateral hemorrhagic lesion.
- Demonstrated clear consciousness, a stable clinical condition, and sufficient tolerance for rehabilitation treatment.
- Had complete assessment data available before and after treatment.
- Baseline FM-UE score ≤ 57, considering that the primary endpoint was defined as a minimum 9-point improvement on the Fugl-Meyer Upper Extremity (FM-UE) Scale (12) (maximum score of 66).

The following participants were excluded:

- Those with hemiplegia or upper extremity paralysis caused by non-cerebrovascular conditions (e.g., traumatic brain injury, brain tumors, Guillain-Barré syndrome, multiple sclerosis, or myasthenia gravis).
- Individuals with severe organ failure, including heart, lung, liver, or kidney dysfunction.
- Those with cognitive impairments that interfered with communication or cooperation.
- Patients with hemi-spatial neglect or hemianopia.
- Individuals with incomplete clinical or assessment data.

### Treatment regiments

#### Pharmacotherapy

All participants received medication treatment according to their medical needs, which included antihypertensives, hypoglycemics, dementia medications, muscle relaxants, psychotropic medication, neuroprotective agents, antineuropathic agents, anticonvulsants, and sedative-hypnotics.

#### Routine rehabilitation therapy

Routine rehabilitation therapy included acupuncture, physical therapy [such as Bobath therapy (13), Brunnstrom therapy (14), Proprioceptive Neuromuscular Facilitation therapy (15) and motor relearning program (16)], together with occupational therapy.

#### HF-rTMS

In this study, HF-rTMS was performed using the MagTD60 transcranial magnetic stimulator produced by Yiruide Company in Wuhan. A circular coil (diameter 125 mm) was selected because its wider stimulation area is more suitable for targeting large cortical regions related to upper limb motor function (17).

In accordance with the recommendations of the International Federation of Clinical Neurophysiology, the resting motor threshold (RMT) of both cerebral hemispheres was determined using single-pulse TMS. The procedure was as follows: Surface recording electrodes were placed on the belly of the contralateral abductor pollicis brevis (APB) muscle, with the reference electrode placed on the tendon and the ground electrode on the wrist. The position of the magnetic stimulation coil was adjusted to identify the optimal location for evoking motor-evoked potentials (MEPs) from the APB muscle in the cerebral cortex (i.e., the motor hotspot). After identifying the motor hotspot, it was marked on the scalp with a pen to ensure consistent coil placement. The stimulation intensity was then gradually reduced, and the minimum intensity required to evoke MEPs with an amplitude greater than 50 μV in at least 5 of 10 stimulations in the resting state was defined as the RMT for that hemisphere (18). If the RMT could not be obtained in the affected hemisphere, the symmetrical point along the mid-sagittal plane from the motor hotspot of the healthy hemisphere was used as the stimulation site for the affected hemisphere.

After determining the stimulation site for the affected hemisphere, the participant was positioned either sitting or supine. The circular coil was placed tangentially to the skull, with the center of the coil positioned anterior to the target site, so that the motor hotspot was located at the edge of the stronger magnetic field. The handle of the coil was oriented at a 45^°^ angle to the mid-sagittal line, pointing to the posterior-lateral direction. The stimulation parameters were as follows: (1) The stimulation intensity ranged from 80 to 100% of the RMT, which ensures patient safety while sufficiently exciting the motor cortex (19). (2) The stimulation frequency was set at 10 Hz, with each train lasting 2 s, followed by a 15-s interval, for a total of 60 trains, resulting in 1,200 pulses per session. The treatment was administered once daily, six times per week (20).

#### Patient grouping

Participants who received HF-rTMS during hospitalization were classified as the HF-rTMS group, while those who did not were classified as the non-rTMS group.

### Outcome measures

#### Primary endpoint measures

The primary endpoint of this study was the proportion of participants who achieved a Minimal Clinically Important Difference (MCID) on the FM-UE scale, defined as an increase of ≥9 points from baseline to the end of the treatment period (12). In line with our clinical routine for discharge evaluation, the post-treatment FM-UE assessment was typically performed within 24 h following the final HF-rTMS session. This timing aligns with the objective of capturing the consolidation of treatment-induced neuroplasticity rather than transient after-effects, as supported by previous rTMS literature reports (21, 22).

The FM-UE Scale is used to evaluate the motor function of the affected upper limb (23), covering 33 sub-items, including reflexes, shoulder, elbow, wrist, and hand movements. Each sub-item is scored on a 3-point scale (0–2 points), with a total possible score of 66 points. Higher scores indicate better functional ability. By calculating the proportion of patients who achieved a 9-point improvement (rather than absolute score changes), this study aimed to identify the proportion of patients who realized significant functional recovery through HF-rTMS during inpatient rehabilitation.

#### Secondary outcome measures

The secondary outcomes include changes in the Modified Barthel Index (MBI) and Brunnstrom Score from baseline to the end of hospitalization, which are functional assessment measures. The MBI (24) is primarily used to assess a patient’s ability to perform activities of daily living (ADL). It consists of 11 items, each graded on a 5-level scale, with a maximum score of 100 points. Higher scores indicate greater independence in daily self-care activities. The Brunnstrom Staging System (25) is used to assess motor recovery in patients with stroke, consisting of six stages, Stage 1: Flaccidity, no reflexes or voluntary movement, muscles are flaccid; Stage 2: Basic Synergies, minimal volitional control, spasticity, and muscle synergies (flexor or extensor) emerge; Stage 3: Voluntary Control, voluntary movement outside synergy patterns, plasticity increases; Stage 4: Coordination, isolated joint movements and muscle coordination, spasticity declines; Stage 5: Complex Movement Patterns, fine motor skills develop; more normal movement patterns; Stage 6: Normal Function, complete recovery with normal motor function, minimal to no spasticity. The stages represent the progression from flaccidity to spasticity and eventually to voluntary, coordinated motor control.

#### Safety issues

Safety issues referred to any adverse reactions potentially associated with HF-rTMS. The doctors who administered HF-rTMS recorded adverse events, capturing incidents that occurred during the treatment and within 120 min after its completion.

### Sample size

The sample size calculation was conducted using Stata SE 13. Assuming that 20% of participants in the non-rTMS group and 35% in the TMS group will reach the primary endpoint, with a Type I error rate (α) of 0.05 and a Type II error rate (β) of 0.05, the required sample size for each group was determined to be 138.

### Statistical analysis

The analysis presented continuous variables as the mean accompanied by the standard deviation (SD) or as the median with an interquartile range (IQR, 1^st^ to 3^rd^ quartile), depending on their distribution. For two-group comparisons, the independent sample Student’s t-test was used for normally distributed data, whereas the Mann–Whitney test was applied for non-normally distributed data. Categorical variables were presented as counts and percentages. The statistical significance of categorical variables was assessed using Pearson’s chi-square test or Fisher’s exact test.

Multivariate logistic regression was conducted to evaluate the two treatment groups concerning the primary endpoints. Based on established statistical suggestions (26, 27) variables with a univariate analysis *p*-value of <0.1 were included in the multivariate logistic regression model to avoid potential omission of relevant predictors. Those variates with clinical meanings related to the primary endpoint were adjusted, ignoring the *p*-value. The results were expressed as adjusted odds ratios (ORs) with 95% confidence intervals (95% CIs).

Overlap-Weighted propensity score matching (PSM) was a propensity score method developed to emulate essential features of clinical studies. It assigned weights to patients according to their probability of belonging to each treatment group, thereby including all patients and achieving an exact balance in the mean of all covariates within the model (28).

All hypothesis tests were two-sided, and statistical significance was defined as a *p*-value < 0.05.

Stata SE 13 (Serial number 401306302851), R software (version 4.3.0, http://cran.r-project.org/), and Prism^1^ were applied for the data analysis.

## Results

**Figure 1:**
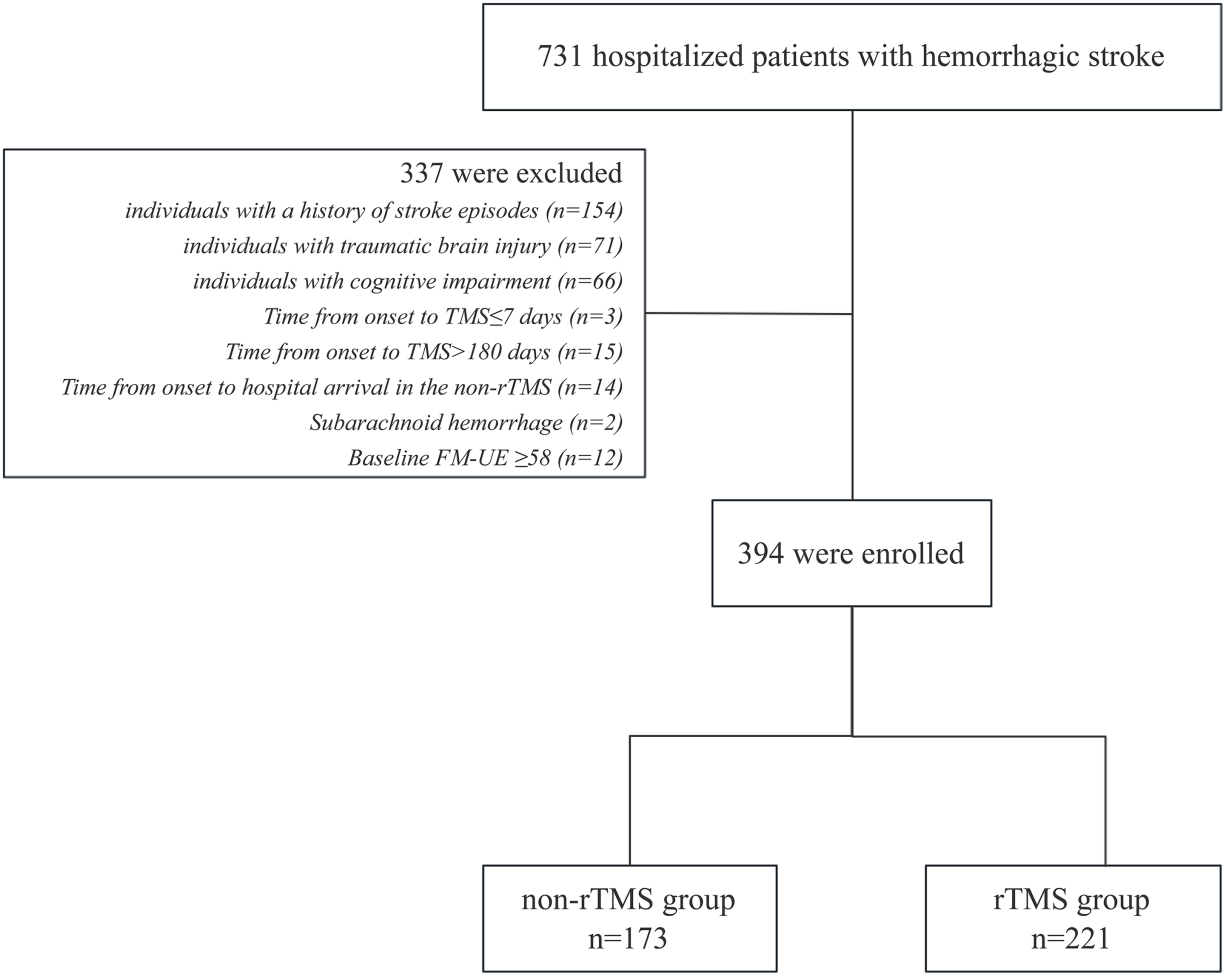
Diagram of participants enrollment.

Out of 731 participants with ICH who sought treatment at our department between January 2020 and June 2023, 394 were included in our analysis. Among these, 173 were categorized into the non-rTMS group, and 221 into the HF-rTMS group (Figure 1).

### Baseline information

Table 1 presents the general characteristics of all participants, comparing the non-rTMS and HF-rTMS groups in both the original dataset and the Overlap-Weighted PSM dataset. In the original dataset, sex distribution differed significantly between the groups (*p =* 0.027), with a higher proportion of females in the non-rTMS group. Other variables, including age, smoking, alcohol consumption, hypertension, diabetes, history of surgery, hospital stay duration, and acupuncture treatment, did not show significant differences between groups (*p* > 0.05). However, the median days between stroke onset and treatment was significantly longer in the HF-rTMS group than in the non-rTMS group (32 vs. 24 days, *p* < 0.001). For stroke-affected brain regions, no significant differences were observed. The proportions of patients receiving medication therapies, including neuro-metabolic enhancers, muscle relaxants, cognitive enhancers, psychotropic medications, pain relievers, and sedatives, were similar between groups, except for the use of cognitive enhancers, which was significantly higher in the HF-rTMS group (*p =* 0.010). The baseline FM-UE scores did not differ between groups.

**Table 1:** General characteristics of all participants.

	Original dataset				Overlap weighted PSM dataset			
	non-rTMS (*n* = 173)	HF-rTMS (*n* = 221)	SMD	*p*-value	non-rTMS (*n* = 88.90)	HF-rTMS (*n* = 88.90)	SMD	*p*-value
Sex (female; %)	66 (38.15)	60 (27.15)	0.236	0.027	28.99 (32.61)	28.99 (32.61)	<0.001	>0.999
Age [mean (sd)]	55.97 (13.57)	54.38 (13.23)	0.118	0.243	54.85 (13.81)	54.85 (13.11)	<0.001	>0.999
Smoke (%)	61 (35.26)	96 (43.44)	0.168	0.123	34.12 (38.37)	34.12 (38.37)	<0.001	>0.999
Alcohol (%)	61 (35.26)	87 (39.37)	0.085	0.465	33.58 (37.77)	33.58 (37.77)	<0.001	>0.999
Hypertension (%)	138 (79.77)	171 (77.38)	0.058	0.653	68.57 (77.13)	68.57 (77.13)	<0.001	>0.999
Diabetes (%)	22 (12.72)	25 (11.31)	0.043	0.787	11.22 (12.62)	11.22 (12.62)	<0.001	>0.999
Surgery (%)	91 (52.60)	134 (60.63)	0.163	0.135	50.17 (56.44)	50.17 (56.44)	<0.001	>0.999
Days in hospital [mean (sd)]	21.00 (6.93)	21.24 (5.78)	0.037	0.713	21.32 (6.84)	21.32 (5.75)	<0.001	>0.999
Days between onset and treatment [Median (IQR)]	24 (15, 44)	32 (24, 58)	0.241	<0.001	26.00 (15.42, 53.00)	31.00 (21.00, 51.00)	<0.001	0.033
Stroke-Affected Brain Region *n* (%)			0.301	0.125			0.305	0.183
Basal ganglia	125 (72.25)	168 (76.02)			65.14 (73.27)	65.29 (73.44)		
Brainstem	3 (1.73)	7 (3.17)			1.50 (1.68)	3.29 (3.70)		
Cerebral cortex	21 (12.14)	19 (8.60)			11.95 (13.44)	7.58 (8.53)		
Subcortical	1 (0.58)	8 (3.62)			0.60 (0.67)	3.54 (3.98)		
Cerebellum	2 (1.16)	1 (0.45)			1.30 (1.46)	0.56 (0.63)		
Thalamus	21 (12.14)	18 (8.14)			8.43 (9.48)	8.64 (9.72)		
Acupuncture (%)	157 (90.75)	203 (91.86)	0.039	0.836	80.74 (90.82)	80.74 (90.82)	<0.001	>0.999
Medication Therapies (%)								
Neuro-metabolic enhancers	93 (53.76)	104 (47.06)	0.134	0.223	44.35 (49.88)	44.35 (49.88)	<0.001	>0.999
Muscle relaxants	31 (17.92)	43 (19.46)	0.039	0.796	16.90 (19.01)	16.90 (19.01)	<0.001	>0.999
Cognitive enhancers	26 (15.03)	58 (26.24)	0.280	0.010	17.01 (19.14)	17.01 (19.14)	<0.001	>0.999
Psychotropic medication	10 (5.78)	10 (4.52)	0.057	0.740	4.69 (5.27)	4.69 (5.27)	<0.001	>0.999
Pain relievers	17 (9.83)	13 (5.88)	0.147	0.203	6.66 (7.49)	6.66 (7.49)	<0.001	>0.999
Sedatives	18 (10.40)	11 (4.98)	0.205	0.064	6.00 (6.74)	6.00 (6.74)	<0.001	>0.999
Baseline score of FM-UE [median (IQR)]	4 (4, 10)	4 (4, 9)	0.081	0.950	4 (4, 11)	4 (4, 9)	<0.001	0.542

FM-UE, Fugl-Meyer Upper Extremity; IQR, interquartile range, 1st to 3rd quartile; PSM, Propensity Score Match; HF, High-Frequency; rTMS, repetitive Transcranial Magnetic Stimulation; SD, standard deviation; SMD, standard mean difference.

In the Overlap-Weighted PSM dataset, all variables were balanced, as indicated by standardized mean differences close to zero and *p*-values > 0.999, except for the days between onset and treatment, which remained significantly different (*p =* 0.033).(Table 1).

The baseline FM-UE scores were similar between the non-rTMS and HF-rTMS groups, with no significant difference observed (*p* = 0.950). The median (IQR) baseline FM-UE score was 4 (4–10) in the non-rTMS group and 4 (4–9) in the HF-rTMS group. The results of FM-UE scores, as well as upper limb function evaluated by MBI and Brunnstrom score at baseline, are presented in Appendix Table 1.

### Primary endpoint

**Figure 2:**
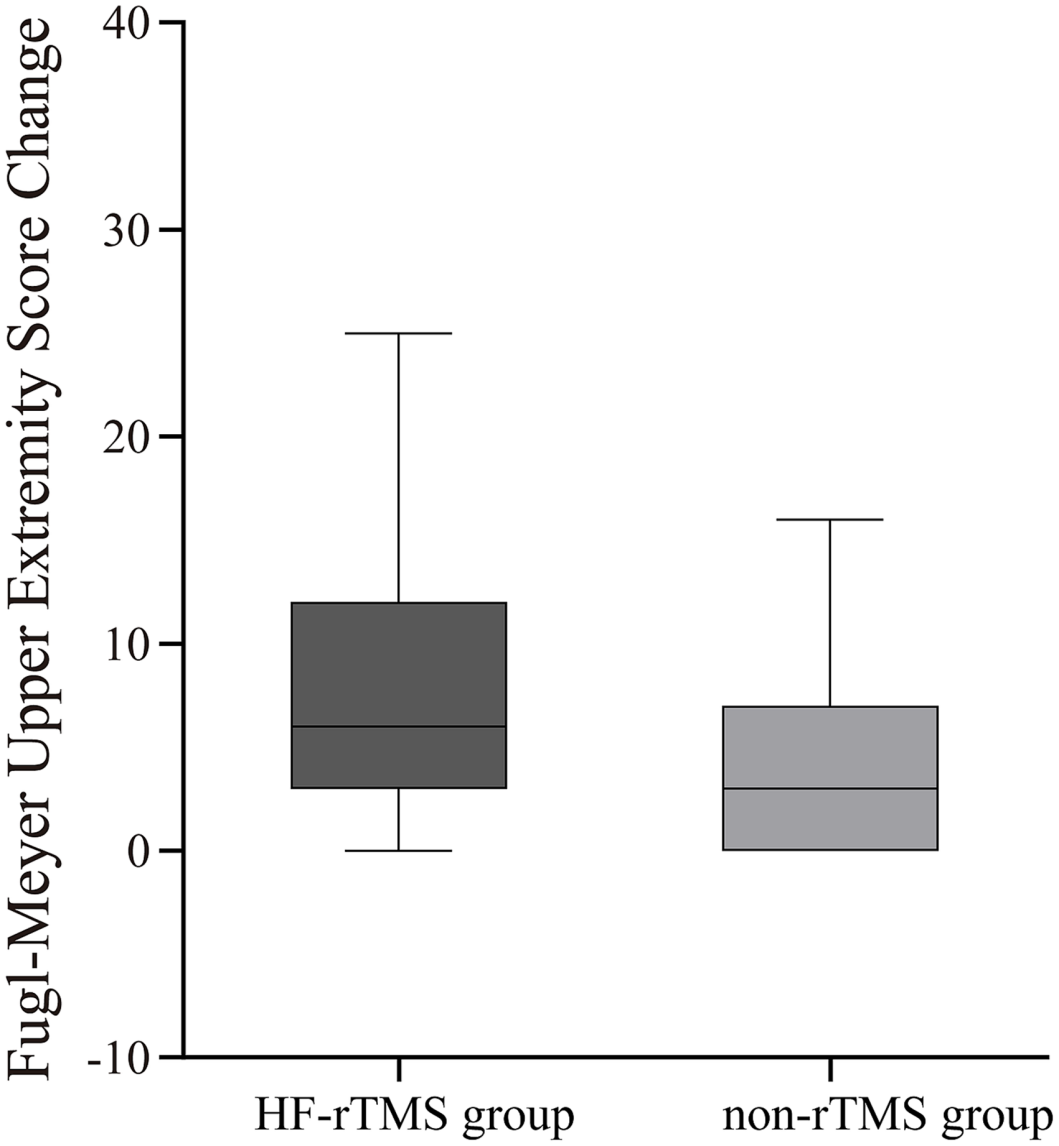
Fugl-Meyer Upper Extremity score change between two groups. The box indicates the interquartile range, with the horizontal line representing the median. The whiskers denote the minimum and maximum values within 1.5 × IQR.

**Figure 3:**
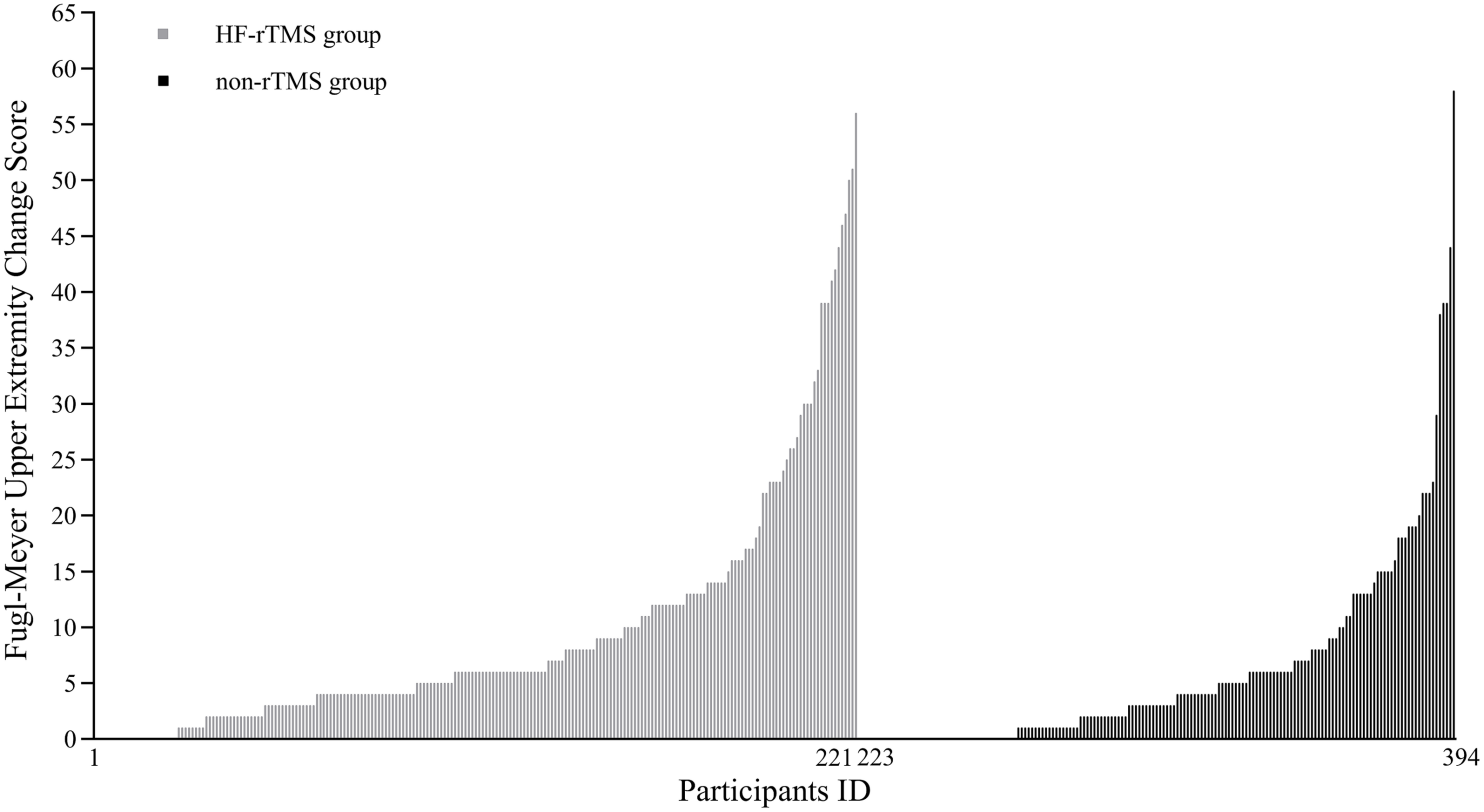
Individual changes in Fugl-Meyer Upper Extremity (FM-UE) scores. Each bar represents the FM-UE score change for a single participant. Bars with zero height indicate no change in FM-UE score before and after treatment. Participants 1–221 correspond to the HF-rTMS group, and participants 223–394 correspond to the non-rTMS group.

The median (IQR) FM-UE score at the end of treatment was significantly higher in the HF-rTMS group (11 [8–29]) compared to the non-TMS group (8 [5–21], *p* = 0.001). The change in FM-UE scores also showed a significant improvement in the HF-rTMS group (6 [3–12]) compared to the non-TMS group (3 [0–7], *p* < 0.001). Additionally, a significantly greater proportion of participants in the HF-rTMS group achieved an FM-UE score change of ≥9 points (34.39%) compared to the non-TMS group (21.39%), with a statistically significant difference (*p* = 0.005; Table 2; Figures 2, 3).

**Table 2:** Treatment efficacy on upper limb function evaluated by FM-UE Scale.

	Non-TMS (*n* = 173)	HF-rTMS (*n* = 221)	Statistics	*p*-value
End FM-UE Score [median (IQR)]	8 (5–21)	11 (8–29)	z = −3.44	0.001
Change of FM-UE Score [median (IQR)]	3 (0–7)	6 (3–12)	z = −4.63	< 0.001
FM-UE Score Change ≥9	37 (21.39%)	76 (34.39%)	χ2 = 8.02	0.005

HF, High-Frequency; rTMS, repetitive Transcranial Magnetic Stimulation; FM-UE, Fugl-Meyer Upper Extremity; IQR, interquartile range, 1^st^ to 3^rd^ quartile.

#### Logistic regression analysis on the primary endpoint based on the original dataset

In the univariate analysis on the primary endpoint (FM-UE score change ≥ 9), several factors were significantly associated with achieving the primary endpoint. Participants older than 60 years had higher odds of achieving an FM-UE score change of 9 or more, (OR = 1.60, 95% CI: 1.02 to 2.50, *p* = 0.040). Participants with a stroke in the brainstem (OR = 5.37, 95% CI: 1.47 to 19.60, *p* = 0.011), cerebral cortex (OR = 2.93, 95% CI: 1.48 to 5.79, *p* = 0.002), and thalamus (OR = 4.17, 95% CI: 2.10 to 8.30, *p* < 0.001) associated with higher probability to get at least 9 points improvement assessed by FM-UE scale compared with those whose stroke in basal ganglia. Patients treated between 30 and 90 days after onset had significantly lower odds of achieving a significant change in FM-UE score (≥9) compared to those treated within 30 days (OR = 0.59, 95% CI: 0.36 to 0.95, *p* = 0.030). Patients treated more than 90 days after onset also had lower odds (OR = 0.68, 95% CI: 0.32 to 1.44, *p* = 0.314). Surgery was associated with lower odds of achieving the primary endpoint (OR = 0.53, 95% CI: 0.34 to 0.83, *p* = 0.005). A baseline FM-UE score greater than 5 was a strong predictor of achieving an FM-UE score change of 9 or more (OR = 4.92, 95% CI: 3.09 to 7.85, *p* < 0.001). Participants in the HF-rTMS group associated with a higher odd of achieving this level of improvement (OR = 1.93, 95% CI: 1.22 to 3.04, *p* = 0.005; Table 3).

**Table 3:** Logistic regression on the primary endpoint (FM-UE score change ≥ 9 points).

	Univariate		Multivariate	
	OR (95% CI)	*p*-value	OR (95% CI)	*p*-value
Sex (female)	1.24 (0.78, 1.97)	0.357		
Age (>60)	1.60 (1.02, 2.50)	0.040	1.42 (0.80, 2.51)	0.229
Smoke	0.90 (0.57, 1.41)	0.645		
Drink	0.83 (0.53, 1.31)	0.428		
Stroke-affected brain region				
Basal ganglia	Ref.			
Brainstem	5.37 (1.47, 19.60)	0.011	1.60 (0.37, 6.97)	0.529
Cerebral cortex	2.93 (1.48, 5.79)	0.002	3.39 (1.53, 7.55)	0.003
Subcortical	2.86 (0.75, 10.97)	0.125	2.31 (0.51, 10.45)	0.278
Cerebellum	0.00 (0.00, inf)	0.986	1.00 (empty)	
Thalamus	4.17 (2.10, 8.30)	<0.001	4.04 (1.76, 9.28)	0.001
Days between onset and treatment (≤30 days as ref.)				
>30 and ≤90 days	0.59 (0.36, 0.95)	0.030	0.35 (0.19, 0.63)	0.001
>90 days	0.68 (0.32, 1.44)	0.314	0.40 (0.16, 0.98)	0.045
Surgery	0.53 (0.34, 0.83)	0.005	0.80 (0.46, 1.39)	0.424
Hypertension	0.96 (0.56, 1.62)	0.866		
Diabetics	1.48 (0.78, 2.81)	0.229		
Acupuncture	1.34 (0.59, 3.05)	0.489		
Medication therapies				
Neuro-metabolic enhancers	1.08 (0.70, 1.67)	0.738	1.06 (0.63, 1.78)	0.836
Muscle relaxants	0.63 (0.35, 1.16)	0.139	0.69 (0.33, 1.42)	0.311
Cognitive enhancers	0.79 (0.46, 1.37)	0.401	0.71 (0.36, 1.40)	0.325
Psychotropic medication	0.61 (0.20, 1.86)	0.383	0.73 (0.20, 2.62)	0.627
Pain relievers	1.27 (0.57, 2.80)	0.558	1.18 (0.45, 3.09)	0.739
Sedatives	0.63 (0.25, 1.59)	0.327	0.75 (0.26, 2.21)	0.608
Baseline FM-UE Score >5	4.92 (3.09, 7.85)	<0.001	5.92 (3.36, 10.43)	<0.001
HF-rTMS	1.93 (1.22, 3.04)	0.005	3.19 (1.81, 5.65)	<0.001
Days in hospital >20	0.85 (0.55, 1.32)	0.471	1.11 (0.65, 1.90)	0.695

CI, confidence interval; FM-UE, Fugl-Meyer Upper Extremity; inf, infinity; OR, odds ratio; ref., reference; HF, high-frequency; rTMS, repetitive transcranial magnetic stimulation.

The multiple regression analysis in Table 3 examines factors associated with achieving an FM-UE score change of ≥9 points. After adjusting for covariates, participants with cerebral cortex involvement had higher odds of reaching this threshold compared to those with basal ganglia involvement (OR = 3.39, 95% CI: 1.53 to 7.55, *p* = 0.003). Similarly, thalamic involvement was associated with increased odds of improvement (OR = 4.04, 95% CI: 1.76 to 9.28, *p* = 0.001). A longer interval between stroke onset and treatment was associated with reduced odds of achieving this improvement, with OR = 0.35 (95% CI: 0.19 to 0.63, *p* = 0.001) for 30–90 days and OR = 0.40 (95% CI: 0.16 to 0.98, *p* = 0.045) for >90 days, compared to those receiving treatment within 30 days. Baseline FM-UE scores >5 were significantly associated with higher odds of achieving this level of improvement (OR = 5.92, 95% CI: 3.36 to 10.43, *p* < 0.001). HF-rTMS treatment was also a strong predictor of functional improvement (OR = 3.19, 95% CI: 1.81 to 5.65, *p* < 0.001). Other factors, including sex, age, history of surgery, and medication use, were not significantly associated with the outcome. Variables such as age, surgery, days in hospital more than 20, and other location of stroke were not associated to FM-UE change ≥ 9 points (Table 3).

The multiple regression analysis without the usage of other medication is listed in Appendix Table 2 which confirmed that HF-rTMS still associated with a higher probability to achieve the primary endpoint (OR = 3.12, 95% CI: 1.78 to 5.45, *p* < 0.001).

#### Analysis on overlap-weighted PSM dataset

Table 4 presents the logistic regression analysis of FM-UE score improvement (≥9 points) based on the Overlap-Weighted PSM dataset. A higher proportion of participants in the HF-rTMS group achieved this clinically meaningful improvement, and the model examined whether HF-rTMS and timing of treatment initiation were associated with this outcome. HF-rTMS was significantly associated with greater odds of improvement (OR = 2.36, 95% CI: 1.18, 4.73, *p* = 0.016). Regarding the timing of intervention, patients who initiated treatment between 30 and 90 days after stroke onset had significantly reduced odds of achieving the improvement compared to those treated within 30 days (OR = 0.42, 95% CI: 0.20, 0.91, *p* = 0.027). However, for those who began treatment more than 90 days post-onset, the odds did not significantly differ from the reference group (OR = 0.55, 95% CI: 0.17, 1.78, *p* = 0.319).

**Table 4:** Logistic regression analysis of FM-UE score change ≥9 based on the overlap-weighted PSM dataset.

	OR (95%CI)	*p*-value
HF-rTMS	2.36 (1.18, 4.73)	0.016
Days between onset and treatment (≤30 days as ref.)		
>30 and ≤90 days	0.42 (0.20, 0.91)	0.027
>90 days	0.55 (0.17, 1.78)	0.319

FM-UE, Fugl-Meyer upper extremity; HF, high-frequency; rTMS, repetitive transcranial magnetic stimulation; OR, odds ratio; CI, confident interval; ref., reference.

### Secondary outcomes

The change in MBI score from baseline to the end of treatment showed a significant improvement in the HF-rTMS group compared to the non-rTMS group (*p* < 0.001). More participants with Brunnstrom stage 5 and 6 in the HF-rTMS group (*p* = 0.005; Appendix Table 3).

### Safety relevant issues

In the HF-rTMS group, 2 (0.90%) of participants experienced headache, 1 (0.45%) experienced dizziness, and 1 (0.45%) experienced dysphoria.

## Discussion

As a large-scale study focusing on the efficacy of HF-rTMS in patients with subacute ICH, this research utilized real-world data analysis to demonstrate that 10 Hz HF-rTMS applied to the M1 area of the lesioned hemisphere significantly improved upper limb motor function (defined as an improvement of ≥9 points on FM-UE; OR = 3.19). The results remained robust in Overlap-Weighted PSM analysis, more participants in the HF-rTMS group reached the primary endpoint (OR = 2.36). More importantly, patients in the HF-rTMS group exhibited higher levels of independence in activities of daily living (as indicated by increased MBI scores) and better progression in Brunnstrom stages (increased proportion in stages 5–6), suggesting multidimensional clinical benefits of this intervention. These findings provide important evidence-based support for the application of HF-rTMS in the subacute phase of ICH.

It is important to acknowledge that, despite the application of overlap-weighted PSM, a residual difference in the time from stroke onset to treatment initiation persisted between the groups, with the HF-rTMS group starting treatment later. Since neuroplasticity is generally heightened in the early phases of stroke recovery, a delayed intervention would typically be associated with a diminished treatment response (29). Thus, this imbalance likely created a bias against the HF-rTMS group. The fact that we still observed a strong and significant benefit of HF-rTMS under these conditions suggests that the true treatment effect may be larger than reported. This interpretation is reinforced by our multivariate logistic regression analysis (Table 3), which adjusted for this variable among others and still identified HF-rTMS as a strong independent predictor of functional improvement (OR = 3.19). Similarly, the Overlap-Weighted PSM model, which incorporated this variable into its weighting scheme, also confirmed the robustness of the primary finding (OR = 2.36, Table 4).

Compared with previous studies, the improvement in FM-UE observed in this study was lower than that reported in the mixed stroke population cited in guidelines (30, 31). This discrepancy may be attributed to three main factors: First, there were significant differences in baseline functional status between study populations (with a median baseline FM-UE score of 4 in this study vs. 22–40 in other studies), suggesting a “ceiling effect” in functional recovery for patients with severe motor impairments. Second, differences in study design may have contributed to this discrepancy. Previous studies largely included patients with ischemic stroke or mixed stroke populations (32, 33), whereas the unique pathological mechanisms of ICH (such as mass effect of hematoma and neurotoxicity of iron ions) may attenuate the neuroplasticity-inducing effects of rTMS (34). Third, differences in the duration of therapeutic assessment may also play a role. In this study, the primary assessment endpoint corresponded to the hospitalization period, with a median duration of 28 days, whereas other studies typically performed follow-up assessments approximately 3 months after discharge (35).

Subgroup analyses revealed two key prognostic factors: hemorrhage location and baseline FM-UE score. Specifically, patients with cortical or thalamic hemorrhage showed better therapeutic outcomes than those with basal ganglia hemorrhage (OR = 3.88). Although our study lacks direct neuroimaging data to confirm the underlying mechanisms, we speculated that hemorrhage in the basal ganglia frequently involves the posterior limb of the internal capsule, potentially causing severe structural disruption of the corticospinal tract (CST) and thereby limiting the synaptic plasticity effects of HF-rTMS (36). In contrast, cortical or thalamic hemorrhages might relatively spare the subcortical white matter pathways, preserving partial CST integrity. This preserved structural foundation could allow for more effective functional compensation when perilesional residual networks are stimulated by HF-rTMS (37).

Moreover, patients with a baseline FM-UE score of ≥5 points were more likely to achieve the endpoint (OR = 5.92), suggesting that even minimal residual motor function may serve as a “critical factor” for rTMS-induced neuroplasticity (10).

Our study provides specific evidence for the efficacy of HF-rTMS applied to the affected hemisphere in ICH. This invites a comparative discussion with the more established evidence for low-frequency (LF) rTMS applied to the unaffected hemisphere. Current international guidelines accord a Grade A recommendation to LF-rTMS for hand dysfunction post-ICH, while the evidence for HF-rTMS remains at Grade B, largely due to a scarcity of dedicated studies in this population (5). The therapeutic rationale for these two paradigms is distinct: LF-rTMS primarily aims to reduce excessive interhemispheric inhibition from the healthy hemisphere, thereby disinhibiting the affected cortex (38). In contrast, HF-rTMS, as applied in our protocol, directly facilitates cortical excitability and promotes use-dependent plasticity in the perilesional tissue of the affected hemisphere (38). These findings of our study suggest that directly targeting the residual functional capacity of the lesioned hemisphere may represent a viable and effective strategy in subacute ICH.

This study has the following limitations: First, the retrospective design cannot completely avoid selection bias (39). Although we employed multivariable logistic regression and Overlap-Weighted PSM to adjust for confounding factors, unrecorded variables (such as family support and rehabilitation training intensity) may still influence the results. Second, the single-center sample may limit the generalizability of our conclusions, necessitating validation through multicenter studies. Third, we excluded patients in the acute phase (<1 week) and chronic phase (>6 months). Moreover, patients in the subacute phase were not further stratified according to the interval from onset to treatment. Notably, current clinical guidelines and rehabilitation practice generally regard the subacute phase as a single therapeutic window, without recommending differentiated interventions based on narrower temporal subdivisions within this period (5).

Future studies are recommended to compare high-frequency stimulation of the ipsilesional hemisphere with low-frequency stimulation of the contralesional hemisphere to identify the most suitable paradigms for different ICH subgroups. It would also be valuable to explore which patients, such as those with cortical or basal ganglia hemorrhage, may benefit more from each approach. In addition, investigating the potential synergy between HF-rTMS and task-specific, robotic, or pharmacologic rehabilitation strategies, as well as its efficacy beyond the subacute phase, may help clarify whether tailored stimulation remains beneficial in promoting recovery during the chronic stage.

## Conclusion

This large real-world cohort study, targeting solely patients with intracerebral hemorrhage, offers important evidence for the therapeutic value of HF-rTMS in the subacute phase of ICH recovery. In light of the limited number of studies specifically addressing ICH, the results contribute meaningful guidance toward individualized neuromodulation-based rehabilitation approaches in this often-overlooked population.

## Data Availability

The raw data supporting the conclusions of this article will be made available by the authors, without undue reservation.

## Footnotes

1
https://www.graphpad.com/scientific-software/prism/
