# Supplementary material for: The efficacy of high-frequency repetitive transcranial magnetic stimulation on upper extremity function in intracerebral hemorrhage: a real-world retrospective cohort study
Source: Front Neurol. 2025 Dec 10;16:1683536. doi: 10.3389/fneur.2025.1683536 (PMC12727436; doi:10.3389/fneur.2025.1683536)
Supplement: Supplementary file 1 [file Data_Sheet_1.docx]

Appendix for HF-rTMS

# sTable 1. Upper limb function at baseline

|  |  | non-TMS (n=173) | HF-rTMS (n=221) | Statistics | *P*-value |
| --- | --- | --- | --- | --- | --- |
| Baseline FM-UE Score | median (IQR) | 4 (4 - 10) | 4 (4 - 9) | z=-0.06 | 0.950 |
| Baseline MBI Score | mean (SD) | 26.36 (19.95) | 27.48 (20.46) | t=-0.548 | 0.584 |
|  | median (IQR) | 25 (10 - 35) | 25 (15 - 40) | z=-0.418 | 0.676 |
|  | min - max | 0 - 90 | 0 - 95 |  |  |
| Baseline Brunnstrom (%) | stage 1 | 105 (60.69) | 127 (57.47) |  | 0.914 |
|  | stage 2 | 36 (20.81) | 44 (19.91) |  |  |
|  | stage 3 | 16 (9.25) | 21 (9.50) |  |  |
|  | stage 4 | 12 (6.94) | 21 (9.50) |  |  |
|  | stage 5 | 4 (2.31) | 7 (3.17) |  |  |
|  | stage 6 | 0 (0.00) | 1 (0.45) |  |  |

HF: High-Frequency; rTMS: repetitive Transcranial Magnetic Stimulation; FM-UE: Fugl-Meyer Upper Extremity;
MBI: Modified Barthel Index; IQR: interquartile range, 1^st^ to 3^rd^ quartile; SD: standard deviation.

# sTable 2. Logistic regression on the primary endpoint (FM-UE Score Change ≥ 9 points)

|  | Univariate | | Multivariate | |
| --- | --- | --- | --- | --- |
|  | OR (95% CI) | *P*-value | OR (95% CI) | *P*-value |
| Sex (female) | 1.24 (0.78, 1.97) | 0.357 |  |  |
| Age(>60) | 1.60 (1.02, 2.50) | 0.040 | 1.45 (0.84, 2.52) | 0.184 |
| Smoke | 0.90 (0.57, 1.41) | 0.645 |  |  |
| Drink | 0.83 (0.53, 1.31) | 0.428 |  |  |
| Stroke-affected brain region |  |  |  |  |
| basal ganglia | Ref. |  |  |  |
| brainstem | 5.37 (1.47, 19.60) | 0.011 | 1.83 (0.44, 7.65) | 0.410 |
| cerebral cortex | 2.93 (1.48, 5.79) | 0.002 | 3.38 (1.54, 7.43) | 0.002 |
| subcortical | 2.86 (0.75, 10.97) | 0.125 | 2.23 (0.50, 9.94) | 0.293 |
| cerebellum | 0.00 (0.00, inf) | 0.986 | 1.00 (empty) |  |
| thalamus | 4.17 (2.10, 8.30) | <0.001 | 3.88 (1.73, 8.69) | 0.001 |
| Days between onset and treatment  (≤30 days as ref.) |  |  |  |  |
| >30 and ≤90 days | 0.59 (0.36, 0.95) | 0.030 | 0.33 (0.19, 0.60) | <0.001 |
| >90 days | 0.68 (0.32, 1.44) | 0.314 | 0.35 (0.15, 0.83) | 0.016 |
| Surgery | 0.53 (0.34, 0.83) | 0.005 | 0.77 (0.45, 1.32) | 0.342 |
| Hypertension | 0.96 (0.56, 1.62) | 0.866 |  |  |
| Diabetics | 1.48 (0.78, 2.81) | 0.229 |  |  |
| Acupuncture | 1.34 (0.59, 3.05) | 0.489 |  |  |
| Medication Therapies |  |  |  |  |
| neuro-metabolic  enhancers | 1.08 (0.70, 1.67) | 0.738 |  |  |
| muscle  relaxants | 0.63 (0.35, 1.16) | 0.139 |  |  |
| cognitive  enhancers | 0.79 (0.46, 1.37) | 0.401 |  |  |
| psychotropic  medication | 0.61 (0.20, 1.86) | 0.383 |  |  |
| pain  relievers | 1.27 (0.57, 2.80) | 0.558 |  |  |
| sedatives | 0.63 (0.25, 1.59) | 0.327 |  |  |
| Baseline FM-UE Score >5 | 4.92 (3.09, 7.85) | <0.001 | 5.91 (3.38, 10.36) | <0.001 |
| HF-rTMS | 1.93 (1.22, 3.04) | 0.005 | 3.12 (1.78, 5.45) | <0.001 |
| Days in hospital >20 | 0.85 (0.55, 1.32) | 0.471 | 1.04 (0.62, 1.75) | 0.884 |

HF: High-Frequency; rTMS: repetitive Transcranial Magnetic Stimulation; OR: odd ratio; CI: confidence interval; inf: infinity; FM-UE: Fugl-Meyer Upper Extremity; ref. reference;

In this model, the usage of other medication were not considered to be analyzed in the multivariable regression.

# sTable 3. Treatment Efficacy on Upper Limb Function Evaluated by MBI, and Brunnstrom Score

|  |  | non-TMS (n=173) | HF-rTMS (n=221) | Statistics | *P*-value |
| --- | --- | --- | --- | --- | --- |
| End MBI Score | mean (SD) | 43.84 (22.97) | 51.63 (21.81) | t=-3.43 | 0.001 |
|  | median (IQR) | 40 (30 - 60) | 50 (40 - 65) | z=-3.37 | 0.001 |
|  | min-max | 0 - 95 | 0 - 100 |  |  |
| Change on MBI Score | mean (SD) | 17.49 (14.86) | 24.14 (17.23) | t=-4.04 | <0.001 |
|  | median (IQR) | 15 (5 -25) | 20 (10 - 35) | z=-4.14 | <0.001 |
|  | min-max | 0 - 80 | 0 - 90 |  |  |
| end Brunnstrom (%) | stage 1 | 62 (35.84) | 42 (19.00) | Fisher test | 0.005 |
|  | stage 2 | 46 (26.59) | 79 (35.75) |  |  |
|  | stage 3 | 30 (17.34) | 48 (21.72) |  |  |
|  | stage 4 | 20 (11.56) | 23 (10.41) |  |  |
|  | stage 5 | 11 (6.36) | 25 (11.31) |  |  |
|  | stage 6 | 4 (2.31) | 4 (1.81) |  |  |

HF: High-Frequency; rTMS: repetitive Transcranial Magnetic Stimulation; MBI: Modified Barthel Index;
IQR: interquartile range, 1^st^ to 3^rd^ quartile; SD: standard deviation.
